# A Portable Phase-Domain Magnetic Induction Tomography Transceiver with Phase-Band Auto-Tracking and Frequency-Sweep Capabilities

**DOI:** 10.3390/s18113816

**Published:** 2018-11-07

**Authors:** Chan Sam Park, Jiyun Jeon, Byungjoo Oh, Hee Young Chae, Kyeonghwan Park, Hungsun Son, Jae Joon Kim

**Affiliations:** 1 School of Electrical and Computer Engineering, Ulsan National Institute of Science and Technology, Ulsan 44919, Korea; chan4517@unist.ac.kr (C.S.P.); ohbj87@unist.ac.kr (B.O.); laplume@unist.ac.kr (H.Y.C.); khpark@unist.ac.kr (K.P.); 2 School of Mechanical, Aerospace and Nuclear Engineering, Ulsan National Institute of Science and Technology, Ulsan 44919, Korea; jyjeon@unist.ac.kr

**Keywords:** magnetic induction tomography, phase-domain sigma–delta modulator, phase-locked loop, transceiver integrated circuit, phase-band auto-tracking

## Abstract

This paper presents a portable magnetic induction tomography (MIT) transceiver integrated circuit to miniaturize conventional equipment-based MIT systems. The miniaturized MIT function is enabled through single-chip transceiver implementation. The proposed MIT transceiver utilizes a phase-locked loop (PLL) for frequency sweeping and a phase-domain sigma–delta modulator with phase-band auto-tracking for a full-range fine-phase resolution. The designed transceiver is fabricated and verified to achieve the measured signal to noise and distortion ratio (SNDR) of 101.7 dB. Its system-level prototype including in-house magnetic sensor coils is manufactured and functionally verified for four different material types.

## 1. Introduction

Magnetic induction tomography (MIT) is an emerging tomographic imaging modality that utilizes alternate magnetic fields to characterize material properties and exterior shapes of target objects. While the primary magnetic field from a transmitter coil is propagated through target objects, the secondary magnetic field at a receiver coil is phase-shifted by eddy currents which are dependent on their conductivities. Target objects can thereby be characterized by analyzing their phase-shifted amounts [1]. Applications include non-invasive medical imaging, such as brain activity monitoring and non-destructive testing (NDT) for material characterization. Most previous studies have focused on experimental methodologies, modeling methods, and analyses, such as Tikhonov regularization methods [2], the magneto-acousto-electrical tomography with magnetic induction (MAET-MI) method [3], vector acoustic source imaging [4], acoustic source analysis [5], and rotational MIT [6] by utilizing measurement equipment, and their actual overall system is too bulky for portable applications. This past research has been concerned with modeling, methodologies, and analysis with a primary focus on measurement and back-end data processing, which are less related to miniaturizing a system.

Phase-domain MIT systems also require high-resolution phase signals because their phase-shifted amounts at the receiver coil are so small that it is not easy to detect simply. Several methods have been proposed to support high-resolution detection of the phase-shifted signal: digital demodulation [7], impedance analysis [8], and I/Q demodulation [9]. However, these methods impose constraints on the system size because a field-programmable gate array (FPGA) board or analyzing equipment are not suitable for portable implementation. In addition, the MIT requires a frequency-sweep operation because it needs to measure phase-domain responses at various frequencies for object characterization [10]. For this purpose, most previous applications have utilized signal generator equipment for frequency sweeps, rather than in the form of an integrated system.

Therefore, in order to expedite the commercialization of portable MIT devices, this work presents a fully-integrated MIT transceiver circuit which features high-resolution accuracy, full coverage of phase input, phase-to-digital conversion and frequency sweep capabilities. A phase-locked loop (PLL) with a multi-phase voltage-controlled oscillator (VCO) [11] is included for a frequency-sweep magnetic-coil transmission, and a phase-domain sigma-delta modulator (PD-ΔΣM) [12] with phase-band auto-tracking and scalable conversion rate is proposed for the high-resolution detection of magnetic phase shifts through target objects. This proposed MIT transceiver is fabricated and experimentally verified to achieve the measured SNDR of 101.7 dB. For system-level feasibility, a portable-sized MIT system prototype, including in-house magnetic sensor coils, was manufactured and functionally proved to provide the NDT characterization on four types of materials: Saline water, distilled water, atmospheric air, and stainless metal.

## 2. Portable Magnetic Induction Tomography System

**Figure 1:**
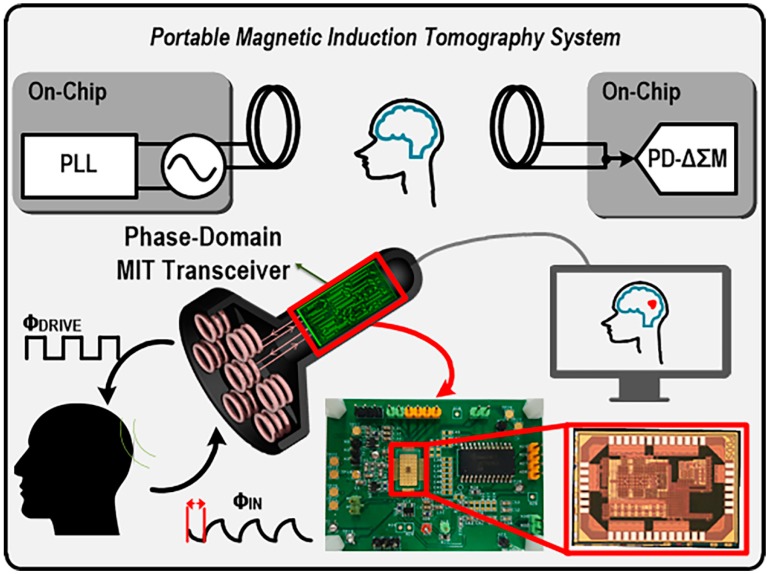
Conceptual diagram of a portable magnetic induction tomography (MIT) system.

A conceptual diagram of the proposed portable MIT system, which consists of magnetic coil sensors, a transmitter with the PLL for frequency generation, and a receiver with the PD-ΔΣM for phase-shift quantization is illustrated in Figure 1. ϕ_DRIVE_ excites the transmitter magnetic coil, then a magnetic fields through target objects propagate to the receiver where eddy currents are induced to give the phase-domain response ϕ_IN_. In this portable MIT system, which has a module size of 6.3 cm × 4.5 cm, various materials are characterized by their inherent phase-shift responses.

**Figure 2:**
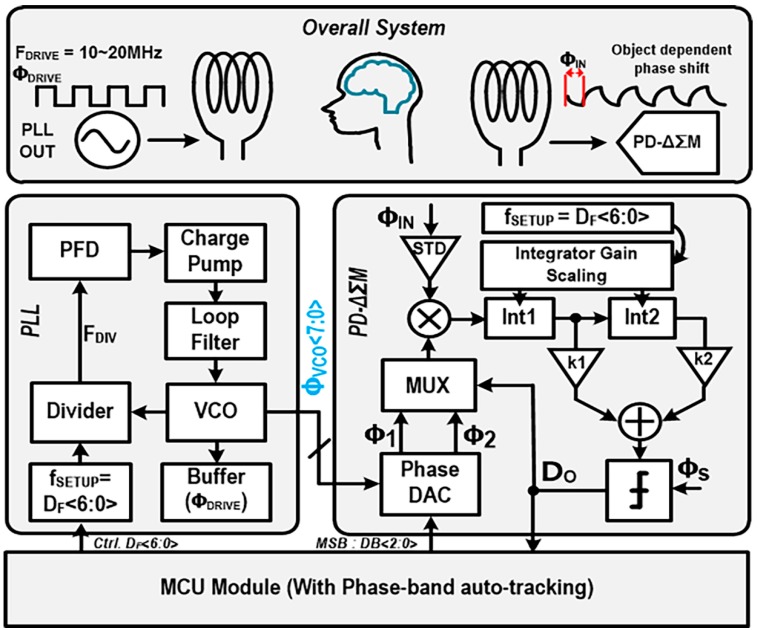
Block diagram of proposed portable MIT transceiver.

The detailed implementation of the proposed phase-domain MIT transceiver which supports a 360-degree full range of phase shifts with a high tomographic resolution of 63 μrad is shown in Figure 2. The transmitter coil is driven by an output buffer of the PLL, its mutual magnetic coupling with the receiver coil is perturbed by target objects that are located in the middle of two transmitter/receiver coils. At the receiver coil, the perturbed magnetic field that possesses the characteristic information on the target objects is converted into a phase-shifted signal, where the eddy current effect at the receiver coil plays a major role. The PLL is designed to have a typical type-II structure and integrates a four-stage ring oscillator for the VCO with eight multi-phase outputs, where the loop filter is designed as 3rd-order to allow sufficient reduction of noise and spurs. The VCO is implemented to cover the frequency-sweep range (10–20 MHz) of the transmitter signal (F_DRIVE_). D_F_<6:0> controls the frequency of the PLL frequency in the transmitter, which is also used to control the integrator gains in the receiver for adaptive operation at different frequencies, this process is programmed by utilizing a microprocessor (MCU). The PD-ΔΣM is implemented with a 2nd-order ΔƩ loop to achieve a relatively high resolution for a given sampling time. In addition, a weighted comparator is utilized to stabilize the integrator output with adaption coefficients (k_1_, k_2_) that are tuned through simulation and stability analysis.

For a 360-degree full coverage of the phase-shift detection, a phase-band auto-tracking scheme is proposed to provide the full-band quantizing capability of the PD-ΔΣM. It detects the phase-band location of the received signal, and then it adaptively controls the phase digital-to-analog converter (DAC). There are some design considerations for the overall MIT system to achieve high-resolution detection of magnetically induced phase shifts. First, the transmit signal should have low-noise and low-distortion performance. For low-distortion performance, the analog PLL is adopted instead of the digital PLL which causes more spurious spurs and distortions. For better noise immunity and even-harmonics rejection, the receiver is designed to include a single-to-differential (STD) amplifier at its front-end and to be fully differential in the remaining signal path. Additionally, a low-noise programmable gain amplifier to provide scalable gain (1–10) for pre-amplifying capability is implemented to enhance the SNR in the receiver front-end path. For noise-shaping or noise-filtering characteristics, the digital conversion is performed by the PD-ΔΣM, which enables a high resolution in phase-to-digital conversion. The PD-ΔΣM itself is also designed to reduce thermal noises in internal integrators and to relax 1/f noise effects in active amplifiers.

## 3. Fully-Integrated Magnetic Induction Tomography Transceiver

### 3.1. Signal Generation with Phase-Locked Loop (PLL)

**Figure 3:**
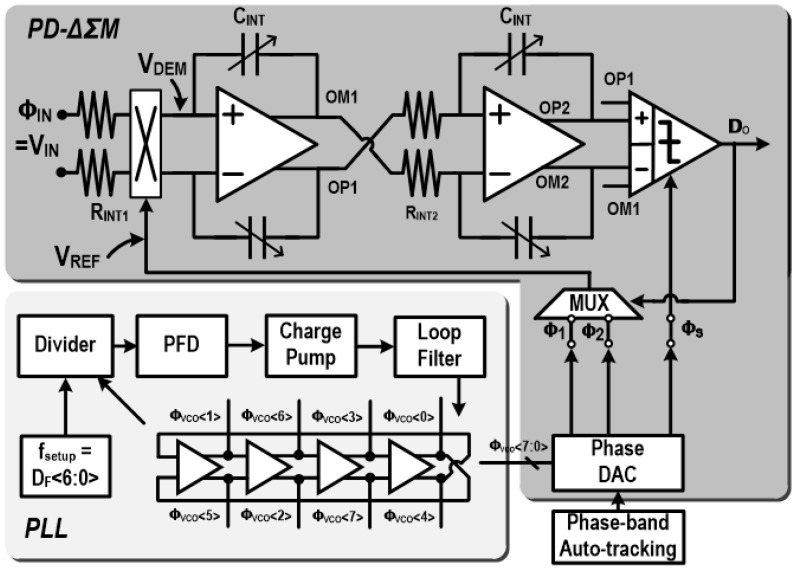
Circuit-level implementation of PLL and PD-ΔΣM.

A circuit-level implementation of the proposed MIT transceiver, which is mainly composed of the PD-ΔΣM and the PLL, is shown in Figure 3. The PLL which provides the frequency-sweep capability for the driving frequency (F_DRIVE_) is implemented as type-II since the type-I can show a potentially unstable steady state, which is undesired for the system. It consists of a phase-frequency detector (PFD), a charge pump, a loop filter, a VCO, and a frequency divider. The PFD detects phase/frequency differences between a reference clock and a divider output, and the charge pump and the loop filter convert the difference information into a control voltage for the VCO. Then, the VCO output is fed back through the divider to the PFD, and the negative feedback operation adopts the VCO frequency to be D_F_ times of the reference clock frequency. As such, the PLL can provide the desired frequency-sweep function by changing the divider value D_F_. The VCO block is designed with a four-stage ring oscillator, where each stage is implemented to be fully-differential for better supply noise immunity. Since the four-stage differential VCO provides eight-phase outputs, the PD-ΔΣM is designed to selectively extract two adjacent phase signals from these eight-phase VCO outputs, which is performed in the phase DAC. For 50% duty-cycled signal generation, eight-phase signals are re-generated by utilizing the rising edges of the eight-phase VCO output signals. This proposed VCO-based reference-phase generation for the PD-ΔΣM removes the necessity of additional blocks and power consumption, and the overall system can be optimized by sharing the VCO block for the transmitter and the receiver. The loop filter is designed to have a 3rd-order structure that consists of two same-valued resistors of 4.174 KΩ, three capacitors of 728 pF, 13 nF and 182 pF. These loop-filter components, especially the capacitors, have large values that cannot be integrated inside the chip, and thus external components are utilized. In order to drive the transmitter magnetic coil, high-voltage output buffers are integrated together.

### 3.2. High-Resolution Phase-Information Detection with Phase-Band Auto-Tracking

The PD-ΔΣM is mainly composed of two integrators, a multiplier, a phase DAC, and a weighted comparator, as shown in the upper part of Figure 3. The receiver input ϕ_IN_ enters the multiplier, and it is multiplied with reference-phase signals (V_REF_), which are commonly derived from the VCO outputs. If these signals are approximated as first-order term in their series expansion for comfortable analysis,

(1) $$V_{\mathrm{IN}}\approx A_{1}\sin\left(w_{DRIVE}t+\phi_{\mathrm{IN}}\right)$$

(2) $$V_{\mathrm{REF}}\approx \frac{4}{\pi }A\sin\left(w_{DRIVE}t+\phi_{REF}\right),$$

where *A* is the supply voltage and *A*_1_ is the input amplitude, $w_{DRIVE}$ is the driving frequency of the transmitter, and $\phi_{REF}$ is ϕ_1_ or ϕ_2_. Then, the multiplier output becomes Equation (3)

(3) $$V_{\mathrm{DEM}}=\frac{4}{\pi }A\sin\left(w_{DRIVE}t+\phi_{ref}\right)\cdot A_{1}\sin\left(w_{DRIVE}t+\phi_{\mathrm{IN}}\right)\;=\frac{2}{\pi }A\cdot A_{1}\left\{\cos\left(\phi_{\mathrm{IN}}-\phi_{REF}\right)+\cos\left(2w_{DRIVE}t+\phi_{\mathrm{IN}}+\phi_{REF}\right)\right\}$$

**Figure 4:**
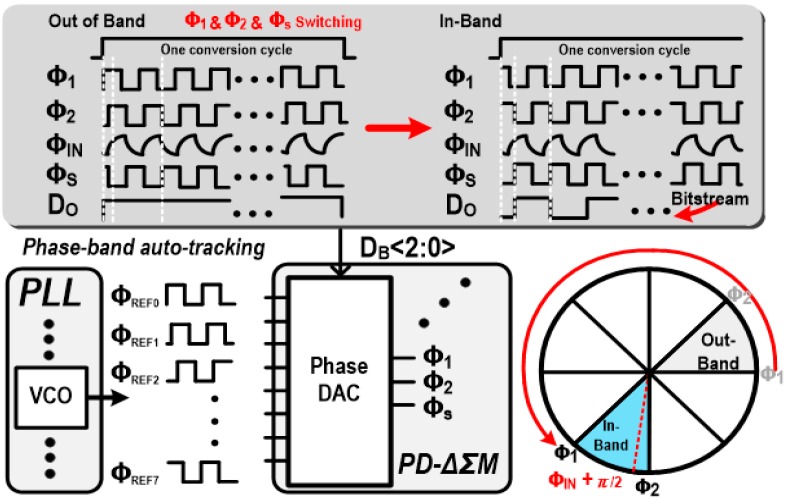
Operational principle of phase-band auto-tracking scheme.

As seen in Equation (3), the multiplier output consists of direct current (DC) and alternating current (AC) components. The following integrators filter out the AC component, and then the DC component can be approximated as

(4) $$\frac{2}{\pi }A\cdot A_{1}\cos\left(\phi_{\mathrm{IN}}-\phi_{REF}\right)\approx \frac{2}{\pi }A\cdot A_{1}\left[\frac{\pi }{2}-\left(\phi_{\mathrm{IN}}-\phi_{REF}\right)\right]\left(@\phi_{\mathrm{IN}}-\phi_{R}\approx \frac{\pi }{2}\right),$$

where $\phi_{R}$ is the average value of $\phi_{REF}$. If $\phi_{REF}-\phi_{\mathrm{IN}}$ becomes around $\frac{\pi }{2}$ by adjusting $\phi_{REF}$, $\frac{2}{\pi }A\cdot A_{1}\cos\left(\phi_{\mathrm{IN}}-\phi_{REF}\right)$ can be linearized as $\frac{2}{\pi }A\cdot A_{1}\left[\frac{\pi }{2}-\left(\phi_{\mathrm{IN}}-\phi_{REF}\right)\right]$ as shown in Equation (4). The main role of the PD-ΔƩM loop [13] is to make this DC component be zero by adjusting occurrences of ϕ_1_ and ϕ_2_, and eventually the received input phase $\phi_{\mathrm{IN}}$ is detected as $\phi_{R}-\frac{\pi }{2}$. While conventional PD-ΔƩMs [12,13] can resolve limited region of the input phase, that is, between ϕ_1_ − $\frac{\pi }{2}$ and ϕ_2_ − $\frac{\pi }{2}$, the designed PD-ΔƩM intends to support 360-degree full coverage by utilizing a proposed phase-band auto-tracking scheme. The proposed auto-tracking scheme is facilitated by using the phase DAC where ϕ_1_ and ϕ_2_ are adaptively selected among eight-phase VCO outputs ϕ_VCO_<7:0> to fit, so that the input phase becomes located between ϕ_1_ − $\frac{\pi }{2}$ and ϕ_2_ − $\frac{\pi }{2}$. The adaptive switching of ϕ_1_ and ϕ_2_ is automatically performed by monitoring the output bit stream of the PD-ΔΣM (D_O_). Figure 4 illustrates the operation principle of the proposed phase-band auto-tracking scheme. Firstly, ϕ_1_ and ϕ_2_ are initialized by default. If their inter-region does not include ϕ_IN_ + $\frac{\pi }{2}$, ϕ_1_ and ϕ_2_ are sequentially moved to the next phase band until ϕ_IN_ + $\frac{\pi }{2}$ becomes located between ϕ_1_ and ϕ_2_. In case of the out-band condition, the D_O_ bit stream shows high only or low only. If the input phase region is well located, the D_O_ bit stream shows bit-change patterns. In this way, the proposed phase-band auto-tracking is performed, and also works like a coarse phase-domain ADC to give the maximum significant bit (MSB) three-bit conversion output of D_B_<2:0>. In addition, for achieving a high phase resolution, inter-phase is set to be $\frac{\pi }{4}$ which can guarantee resolution. Narrowing the inter-phase region would increase phase resolution, until the input referred noise of the PD-ΔƩM becomes higher than the quantization noise. 

**Figure 5:**
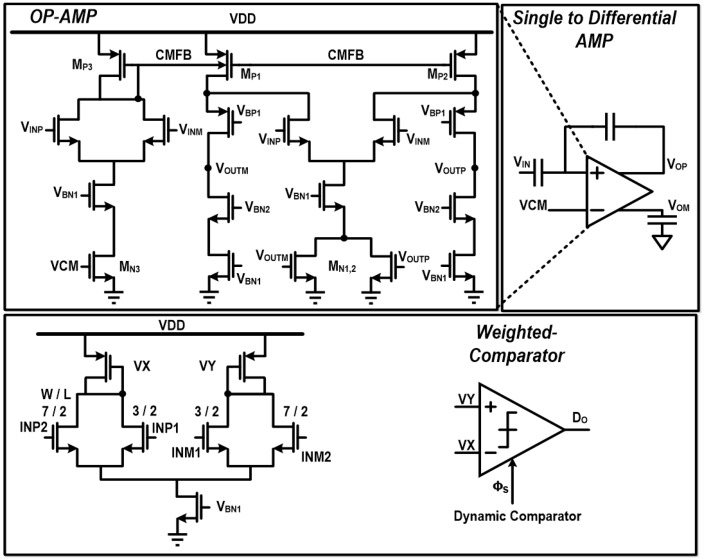
Weighted-comparator and single-to-differential amplifier circuits.

There are some design issues in this MIT transceiver system. Firstly, it should support a frequency-sweep capability to give phase-shift information at different frequencies. As the transmitter frequency changes, the PD-ΔΣM loop might become unstable due to the integrator gain variation, which is caused by the frequency change. Thus, in this work, an integrator gain-scaling function was implemented to guarantee the loop stability by making C_INT_ programmable from 8.5 pF to 18.5 pF. Secondly, internal integrators in the PD-ΔΣM should be designed to not corrupt the SNDR for high-resolution performance. For this purpose, the resistor in the first-stage integrator R_INT1_, which is a dominant noise source, is lowered as 5 KΩ while the second-stage resistor R_INT2_ is 75 KΩ. In addition, an operational amplifier (OP-AMP) in the integrator is designed to have at least twice the bandwidth of the input signal frequency. This OP-AMP circuit is shown in Figure 5, where the gain is 80 dB for high-resolution and the gain-bandwidth product is 40 MHz with 18.5 pF load capacitors. For lower distortion and stable common-mode output level, the designed OP-AMP includes two kinds of common-mode feedback (CMFB) circuits [14] that are implemented with M_N1,2,3_ and M_P1,2_. Thirdly, the PD-ΔΣM is designed to have a 2nd order structure, and it needs to stabilize internal integrators to not be saturated. For this purpose, the proposed PD-ΔΣM adopts a weighted differential comparator which utilizes weighted combination of two internal integrators’ outputs to generate the PD-ΔΣM output (D_O_) [15]. The weighting function is implemented by adding a combining circuit in front of the comparator, where input transistor size ratio decides the weights of two inputs. In this way, coefficients of k_1_ and k_2_ in Figure 2 are designed to be 0.3 and 0.7, respectively.

## 4. Measurement Results

**Figure 6:**
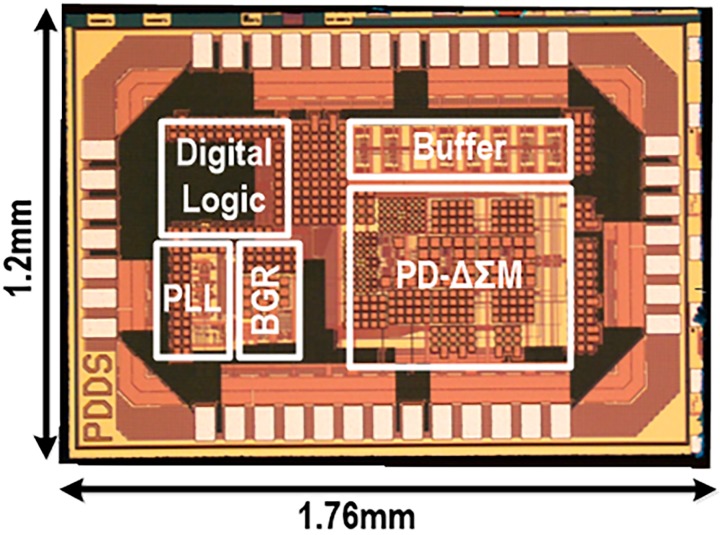
Chip microphotograph of the portable phase-domain MIT transceiver.

**Figure 7:**
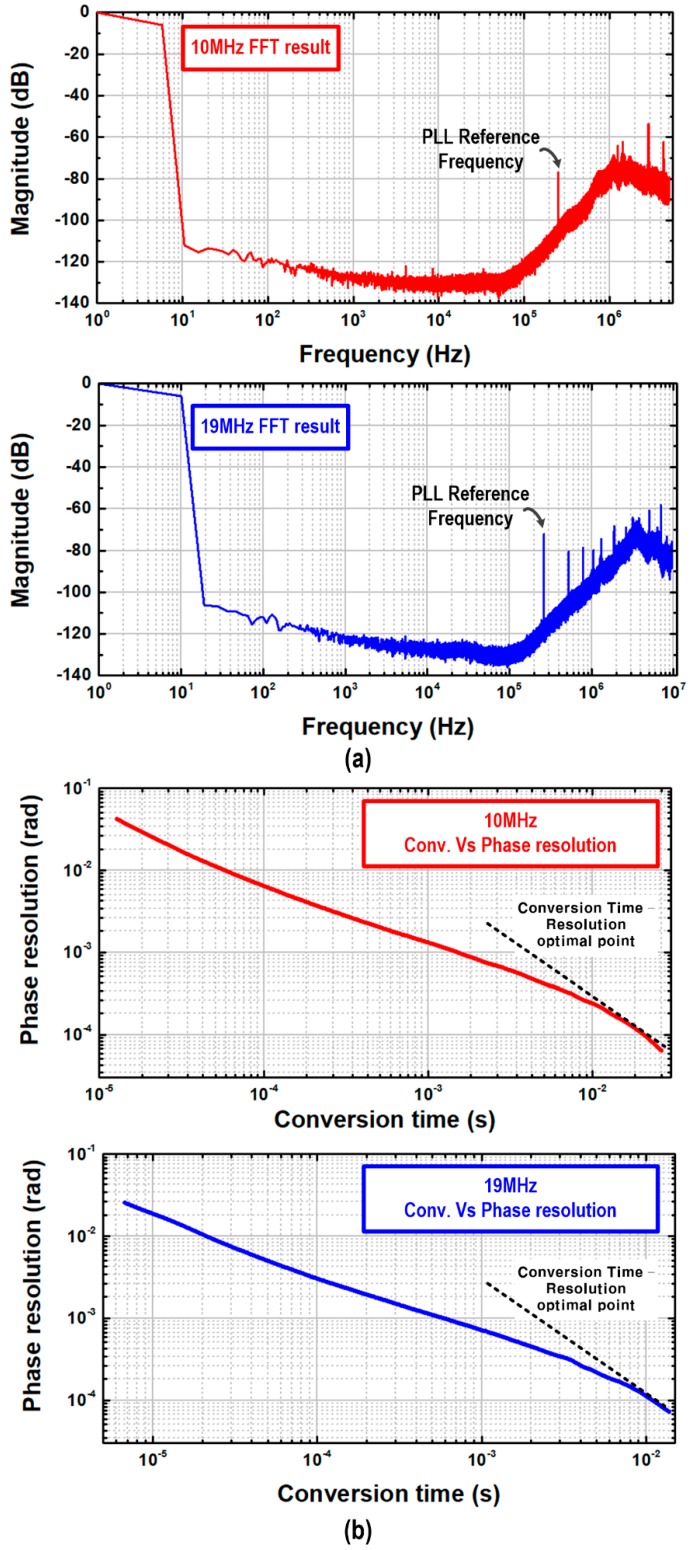
(**a**) Measured fast fourier transform (FFT) plot of phase-domain sigma-delta modulator and (**b**) measured characteristic plot on phase resolution versus conversion time.

A chip prototype of the proposed portable MIT transceiver was fabricated in a 0.18-μm complementary metal-oxide-semiconductor (CMOS) process, and its microphotograph is shown in Figure 6, occupying a silicon area of 1.76 mm × 1.2 mm. This integrated MIT transceiver includes the PLL, its output buffers, and the PD-ΔΣM. The PLL was designed to support the transmitter frequency range from 10 MHz to 20 MHz and also to provide eight-phase signals through the four-stage differential VCO. In addition, its output buffers were integrated together to drive in-house manufactured MIT coils. Then, MIT phase-shifted signals were converted to digital codes in the PD-ΔΣM. Based on this transceiver chip, a portable system prototype with in-house MIT coils was manufactured for its functional feasibility. With a 1.8 V supply, its total current consumption of the MIT transceiver chip was 8.38 mA, where the PLL consumed 4.96 mA and the PD-ΔΣM consumed 3.42 mA. For measurement flexibility, the decimation filter for the PD-ΔΣM was implemented off-chip, and its final digital output was generated from the auto-tracking output and the decimated PD-ΔΣM output. If an object between the transmitter and the receiver coil is defined, the phase shift signal concerned with the object is fixed to DC. Therefore, the signal does not need any high bandwidth of the phase-digital converter. In addition, a signal would appear at the frequency around DC. Figure 7a shows measured FFT waveforms of the PD-ΔΣM at different PLL frequencies of 10-MHz and 19-MHz. They include DC phase signals below 5 Hz and 10 Hz, respectively, and PLL reference spurs at 250 kHz, 500 kHz and 750 kHz appears, which would be filtered out through the decimation process, a kind of digital low pass filter. It can be seen that 1/f noise effects are bigger at the 19-MHz driving condition, but the noise floors are almost the same at both the driving frequencies. This means that the PD-ΔΣM structure has a sufficient noise-shaping capability to guarantee a low-noise low-distortion operation. Figure 7b shows measured characteristic plots of phase resolution versus conversion time. The PD-ΔΣM with optimal resolution performances achieved a reasonable speed around 15-ms conversion time for 10-MHz driving frequency condition and 8-ms conversion time for 19-MHz driving frequency condition.

**Figure 8:**
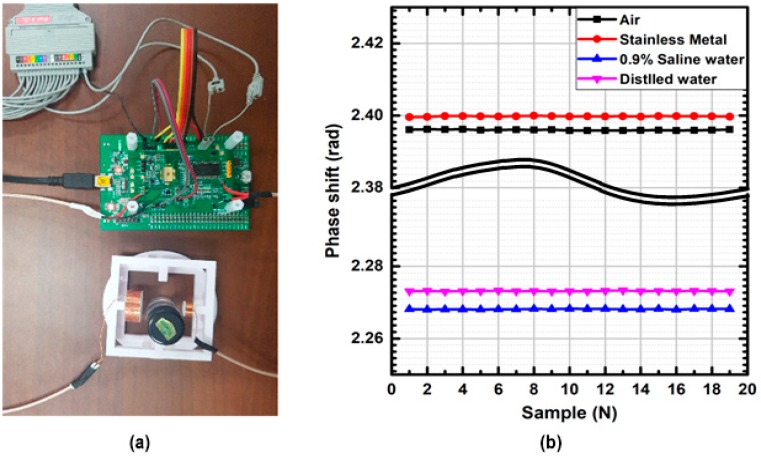
(**a**) Experimental snapshot of proposed phase-domain MIT system prototype and (**b**) measured characteristic plot of phase shift with different material types.

Figure 8a shows a portable phase-domain MIT system prototype with in-house magnetic coils, which consists of a magnetic coil sensor and a transceiver chip-on-board, including the ARM Coretex-F4 MCU for its control and data interface. Figure 8b shows its measured characteristic plot on phase-shift amounts, depending on target material types. With different materials located between transmitter and receiver coils, their measured phase-shift data were obtained from the digital outputs of the MIT receiver, where distilled water and saline water give relatively low phase shifts. While conventional MIT systems have utilized commercial measurement equipment, such as network analyzers or impedance analyzers, this designed transceiver and its system prototype is supposed to replace conventional bulky equipment, and it would contribute to portable-form commercialization of emerging MIT technologies. Table 1 summarizes the overall performance of the proposed MIT transceiver chip, which is also compared with previous work on MIT systems or PD-ΔΣMs. The proposed portable MIT system achieved measured 101.7 dB SNDR, 63 μ-rad phase resolution with 15-ms conversion time for 10-MHz driving frequency condition, and 95.73 dB, 125 μ-rad phase resolution with 8-ms conversion time for 19-MHz driving frequency condition, which is comparable to state-of-the-art technologies.

## 5. Conclusions

A phase-domain MIT transceiver with phase-band auto-tracking and frequency-sweep capabilities was proposed and its system-level feasibility was experimentally verified with a miniaturized system prototype. It was designed to support the full coverage of phase-shifted input through the phase-band auto-tracking algorithm and to support the frequency sweep for various MIT characterizations. The designed transceiver prototype was fabricated and its MIT functionality was achieved through utilizing in-house magnetic sensor coils. This proposed miniaturized work could contribute to the commercialization of portable MIT devices and replace conventional expensive, bulky, equipment-based measurements. MIT imaging applications could also be supported by a simple multi-channel implementation of this transceiver.

## Figures and Tables

**Table 1:** Performance summary and comparison table.

	This Work		[7]	[8]	[12]
**On-Chip Readout**	Y		N	N	Y
**System**	MIT		MIT	MIT	CO_2_ Sensor
**Method**	PD-ΔΣM + PLL		Demodulation + A/D Conv.	Impedance analyzer + Sig gen.	PD-ΔΣM
**Technology**	0.18 μm		N/A	N/A	0.18 μm
**Phase range [rad]**	2*π*		2*π*	2*π*	*π*/15
**Power Consumption [W]**	15.084 m		N/A ***	N/A ***	6.8 m
**SNDR [dB]**	101.7	95.73	59.3 *	79.65 **	N/A
**Conv. time [s]**	15 m	8 m	10 m	3 m	1.8

* Signal to noise ratio (SNR) is extracted. ** From equipment-measurement, SNDR is extracted. *** Reference equipment used. (Signal generator and impedance analyzer or FPGA).
